# An Albertian View of Buchanan’s Contractarianism

**DOI:** 10.1007/s41412-022-00127-6

**Published:** 2022-07-15

**Authors:** Geoffrey Brennan, Hartmut Kliemt

**Affiliations:** 1 Justus Liebig Universitat Giessen, Giessen, Germany; 2 Australian National University, Canberra, Australia

**Keywords:** Critical rationalism, Contractarianism, Value-neutrality of economics, Value-neutrality of economic advice, H. Albert, Buchanan, Sugden, Vanberg, A 12, B 41, D 61

## Abstract

This paper in honor of Hans Albert ‘@100’ seeks to show how adhering to critical rationalist ‘economic philosophy’ avoids contradictions in James Buchanan’s contractarianism: restricting constitutional economic advice to what serves the ends of *all* potential addressees simultaneously Buchanan not only blurs the borderline between value-neutral economic philosophy and substantive moral philosophy but also contradicts his thesis of the “necessary relativism and individualism of values”. Translating ‘means-ends’-relations into technological ‘cause-effect’-relations, Albert can treat technological blueprints as nomological hypotheses subject to scientific test and corroboration while leaving their practical implementation to citizens whose contingent particular ends may or may not be universalistic.

## Introduction and overview

Hans Albert’s critical rationalist contributions to economic methodology are manifold (see Albert 1998). But two things that stand out are his concept of a ‘technology’ and his application of that concept to a wide variety of social practices – including the ‘scientific’ practices of economists.^1^ Technological descriptions can be implemented in practice; but a technology as such cannot determine whether it *should* be implemented; or, where some technology is as matter of fact ‘embodied’ in some practice, whether that practice should be sustained in cases where human actors can intentionally influence that practice.

Each intentional implementation of a technological blueprint is an ‘experiment’ in itself. That experiment can be empirically assessed in a value-neutral manner in terms of whether the combination of nomological hypotheses represented and/or implied by the technological *description* are corroborated by its predicted effects or not. Obviously, all the familiar distinctions between causality and correlation come into play here, as do methods such as randomized controlled trials, time series and econometric analyses. Though employing these methods can be seen as expressive of Hume’s aim of introducing “experimental methods to moral subjects”,^2^ we do not intend to discuss the Humean program of ‘social science’ in general here. For our purposes, it is essential to note that from Hans Albert’s critical rationalist-cum-realist point of view, all these activities can and ought to comply with the methodological norms of standard scientific practice – in particular the requirement of value-neutrality.

The economic philosophy characterization of the process of critical rationalist examination of technologies amounts itself to a technology which is to be assessed according to its own critical rationalist standards (in terms of its likely empirical consequences for pursuing certain ‘hypothetically’ presumed ends of evidence-based disciplinary practice).^3^ In this conceptualization, Albert’s technology concept may be seen as the benchmark in economic philosophy: like disciplinary science, economic philosophy should subject itself to norms of value-neutrality when exploring (institutional) means that may be conducive to human pursuits (whichever these may be).

Buchanan appears to accept the spirit of technological analyses; but he thinks that he can add elements to the technology benchmark without violating basic methodological precepts of value-neutrality. However, once he starts to spell these additions out in terms of his contractarian approach, it becomes clear that his intended restriction of technological analyses to *universally* acceptable technologies is inconsistent with value-neutrality. Buchanan’s contractarianism cannot be accommodated within a conception of economic advice-giving that respects the requirement of value-neutrality as a constraint on scientifically legitimate argument.

Maybe there are other additions that do not fail to comply with value-neutrality, but we will focus here on illustrating why Buchanan’s contractarian universalism is not consistent with a modern critical-rationalist/realist scientific economic practice as spelled out by Albert. Instead of talking *about* Hans Albert, we will rather practice what he preaches; and adopt his technological perspective in criticism of Buchanan’s aim of deriving a universalist foundation of his normative contractarianism within economics.^4^ The present essay argues that incorporating universalist quasi-Kantian contractarian ethical principles of ‘Buchantianism’ (Bu-Kantianism) into precepts of *what economists should do* (Buchanan 1964/1999, vol. 1, pp. 28–42) violates other values to which Buchanan and his followers declare allegiance: Buchantians cannot coherently claim to respect the sovereignty of envisioned advisees and then restrict their advice-giving practice exclusively to technologies that are to the advantage of all potential advisees.^5^

To put this central point slightly differently, Buchanan and his followers do not fully subscribe to the norm that economic advisors ought to be partisan for the particular interests of particular advisees. Buchantian economic advisors would in this regard deviate not only from the role-obligations of other counselling professions as traditionally perceived (the medical and legal professions exemplify) but also from Buchanan’s declared aim of respecting the sovereignty of individual advisees in setting the agenda: if advisees are not asking for what serves the interests of all potential advisees symmetrically, insisting that advice should exclusively focus on what serves the interests of *all* rather than the interests of particular advisees who may not themselves be universalists, is violating Buchanan’s own mantra not to ‘play at being God’ (Buchanan 1975/1999, vol. 7, p. 4).

A critical rationalist ‘Albertian’ technologist whose analysis can serve as advice to sovereign individuals — ones who ‘give’ for themselves the ends, aims and values to be pursued — can avoid any such incoherence. She can focus on the empirical suitability of technologies in a value-neutral way, while remaining silent on whether the ends to which the technologies may be put should or should not be pursued. Under this neutrality condition, providing technological information to those who as a matter of fact want to use that information to serve the ends of *all* is perfectly compatible with having those who use the technological information to serve their own individual purposes regardless of whether this affects others negatively or not.

In the next Sect. (2.) we will briefly explore where economics may be located “between predictive science and moral philosophy” in Buchanan’s scheme of things and beyond; and then turn to different types of norms that might be relevant in economic advice-giving (3.). Next, we expand on our diagnosis that Buchantianism seems to miss the conceptual distinction between particularistic and universalistic individualism (4.). In conclusion we will offer a few general summary observations (5.).

## Economics — between predictive science and moral philosophy

Buchanan accuses economists who do not respect what he calls the “relativism and *individualism* of values” (1986/1999, vol. 17, 170, italics are Buchanan’s) of “playing at being God”. Although in some circles and in relation to certain aspects, imitating God might be an object of approval, that is not the spirit in which Buchanan offers the remark. For him, playing God is a cardinal sin (if that were a category he admitted). With his endorsement of ‘relativism’, Buchanan follows the crowd of economists who tend to subscribe to a narrow conception of reason as instrumental rationality in pursuit of ends that are exogenous to reason.^6^ In consequence, though otherwise mostly critical of Lionel Robbins’ (1933/35) account of the ‘nature and significance of economic science’^7^, Buchanan must be read as concurring with Robbins when it comes to the Weberian ideal of ‘value-neutral science’.

Buchanan clearly thought that ‘economic philosophy’ – a title that he himself used later in life when announcing one of his standard graduate courses – lies somewhere “between predictive science and moral philosophy” (as the title of Buchanan 1987 runs).^8^ Taking the term literally, it seems plausible to think of ‘economic philosophy’ as an extended effort to apply basic economic methods and insights to philosophical issues.^9^ It is here that Hans Albert’s conception of a critical rationalist approach to classical issues of social order (in terms of alternative technological solutions) and Buchanan’s contractarian economic philosophy meet on common ground -- and, being on common ground, it is here that they not only can ‘meet’ but also ‘clash’.

One entry point into Buchanan’s views about what exactly he is trying to do lies in his attitudes to the economist as advisor. That the economist has such an advisory role involves two presumptions: that the economist’s location in the broader division of labor gives her access to knowledge and insights that others do not possess; and that that knowledge and those insights can be ‘useful’ (in some sense yet to be explored).

In a critical rationalist framework, we can characterize Buchanan’s position on economic advice-giving as follows:

1. value-neutrality and respect for individuals’ sovereignty constrain the economist in the role of an advisor (who speaks with scientific authority) to provide technological descriptions that indicate in terms of predictive (explanatory) science whether and how potential ends of those sovereigns may be reached (‘nomological empiricism’);
2. economists *should* restrict their technological descriptions ‘exclusively’ to ‘inclusive’ institutions that further the ends of *each and every* member of a community of individual addressees (‘contractarian universalism’);
3. individuals are to be treated as ‘sovereign’ in setting the ends (for which the advisor merely isolates means) and in implementing the technological means; that is, the economist as advisor must respect not only the advisee’s ends but also the advisee’s agency in securing those ends (‘non-imposition of aims, ends or values and actions’).^10^

Requirements (1) and (3) serve to prevent the economist from deploying her own personal ethical judgments in the assessment of ends: they restrict advice to focusing on *means* to ends that are specified by the advisees as sovereigns and demand respect for the separate advisees’ agency as well as their desires/preferences. As such (1) and (3) are compatible with advising particular individuals on how to pursue particular ends in ways detrimental to the particular ends of other particular individuals.^11^ This, however, contradicts (2) which requires economists to focus *exclusively* on inclusive institutions that further the ends of *each and every* member of a community of individual addressees.

Constraining advice to such ‘Pareto-inclusiveness’ is not compatible with respecting the values of ‘sovereign’ individuals *whatever* those values may be. On the contrary, it excludes those seeking non-inclusive advice by the restriction that advice is ‘properly’ offered only in securing those values of individuals that can be simultaneously furthered for all. Whatever its merits may be, this restriction is inconsistent with treating individuals as *sovereigns* who set the advice-giving agenda for the advisors (being sovereign interpreted as amounting to being normatively unrestricted).

To illustrate, assume for the sake of specificity that there are individuals A, B, C asking successively for advice. The advisor will provide advice concerning the means to the ends of A, then she will provide advice on how B can reach his ends; and then she can provide advice on how C can reach his particular ends. If the technology T that serves the given ends of A will, upon implementation, negatively affect the fulfilment of B’s ends, then this is not a value-neutral reason for the advisor to withhold the information on T from (A) Without a value judgment that implicitly censors the aims, ends or values of A whenever they thwart the ends of another, there is at least no systematic value-neutral reason why the economic advisor should not pass on to A the technological information which A can use at the expense of (B) In short, a critical rationalist like Hans Albert does not encounter the incoherence of Buchanan’s twin aims of subscribing to value-neutrality and contractarian universalism in economics, though he may want to critically examine the effects and conflicts that may result from efforts to realize the technological advice provided to separate advisees.

## Different types of norms in economic advice practice

It may be useful at this point to distinguish three kinds of norms that occupy the space of Buchanan’s various discussions:

(4) ‘purely methodological norms’ — norms that the ‘scientific method’ imposes to establish (or reject) claims about the causal structure of the various propositions that make up a discipline. Such norms involve procedures for the assessment of evidence — for establishing the truth of the (probabilistic) law-like ‘for all …: if X then Y’ claims that constitute the nomological core of ‘science’.
(5) ‘substantive ethical norms’ and judgments about the ‘proper ends’ of human action — and it is mainly these latter judgments that Buchanan’s strictures on ‘playing God’ are designed to eliminate from science.
(6) ‘professional norms’ — norms that are imposed (or understood to be imposed) by being a member of a particular profession. These ‘professional norms’ may refer to methodological norms either directly or by implication but they typically go beyond ‘purely methodological norms’ (in particular when it comes to advice giving).

To illustrate the ‘workings’ of the three types of norms (4)-(6), it is perhaps useful to look at what is going on in the so-called ‘applied sciences’ that are today concentrated at ‘schools’ and ‘polytechnics’. The technological information created by these institutions is meant to be of potential use for *all* ‘cases’ of a certain ‘type’. As types, cases are characterized in terms of general predicates and in that sense may be relevant for anybody to whom they concern in view of her specific aims, ends or values.^12^ In this spirit a (political) economist who is, say, employed at a business school or a university, can generate technological information for all to whom it may concern in pursuit of their particular ends.

Yet, when it comes to personal relations to personal clients in advice-giving more may be involved. For example, medical doctors routinely take the Hippocratic oath. That oath commits those who swear it to certain ends — to the preservation of life and the good health (however exactly defined) of their own personal patients. Moreover, it is of interest here that the classical oaths were strictly focused on the relation to the personal patients of medical doctors rather than on any universalist conception of the common weal. The medical doctor swore that she would be partisan for her personal patient.

This plays out in a public health context. For instance, in the covid-19 pandemic, medical doctors who stick to traditional principles will seek to recommend vaccination if and only if, for a patient with a specific personal risk profile, the expected medical benefits will outweigh the potential harms to health for *this* patient. Collectively desired ends like, say, herd-immunity are not considerations that are supposed to bear in the advice the medical doctor provides in relation to her personal patient.

Though the general proclivity to politicize/collectivize all spheres of life has led to an erosion of the old Hippocratic principles, many medical doctors would still insist that, given the ‘medical’ aims, ends or values which they have sworn to pursue, they are in their role as personal advisors of specific patients not entitled to pass interpersonal priority judgments. For instance, in cases in which the personal risks of adverse side-effects of vaccination are not sufficiently known or are known to outweigh the personal benefits for young individuals each taken separately, the advice to get vaccinated cannot be justified in conformity with the classical interpretation of the obligations of clinical doctors towards their personal patients. Even in cases in which herd-immunity is desirable from the point of view of the common weal (say as defined in utilitarian welfare economic terms) the medical doctor – who sticks to the classical oath – could justify the advice that each and everybody should get vaccinated only if this were in the particular interest of each and everybody separately.

A more dramatic example is organ allocation by medical doctors in the times before allocation algorithms came into use as constitutional rules of allocation. There were many doctors complaining that they could not in compliance with their role obligations weigh patient A’s medical prospects against patient B’s prospects and then say that perhaps B rather than A should get an available organ. These doctors were fully aware that in traditional medical ethics, the requirement of being partisan for a particular individual would prevent giving such impartial advice.

But are the two preceding examples not also examples of a variant of the demand of Pareto-inclusivity and in that sense grist for Buchanan’s mill? After all, it seems that in the preceding cases, public policy advice can be given within the constraints of the partiality requirements of medical ethics *only* if following it will be in the interest of all individuals separately. Yet now, instead of advice concerning what individual patients should do in their separate capacities, advice on a public health policy level is at issue. Sovereign patients who consult their personal clinician as a rule decidedly do not demand to receive advice on which medical treatment would serve the common weal but advice that serves their personal weal. The patients want a medical doctor who like the attorney is partisan for their interest and not the impartial ‘public health judge’ who weighs each patient’s wellbeing against that of others.

As ‘sovereign’ decision-makers, patients seem entitled to make an autonomous decision in relation to their personal health rather than receive a prescription that tells them when to desist from their own interest in favor of that of other persons. Patients expect doctors to employ their superior knowledge of facts in ways partisan for the interests of their personal patient. They mostly expect the medical doctors to live up to the methodologically norms of science and to respect the autonomy of the patient as author of the ends of medical interventions based on this science.

Though medical doctors are notoriously bad in accepting a critically rationalist stance towards their own convictions and their practical judgments concerning treatment decisions, they nowadays seem to accept that their professional role requires such a stance. It includes as an upshot a commitment to ensure that any advice given to a particular patient is based on the best available medical evidence applicable in case of that patient — that patient’s particular aims, ends or values and her diagnosis. In this sense the professional norms presuppose the methodological ones, but they are not themselves methodological norms.

In the economist’s analogue, Buchanan cannot help himself to claims about the content of professional norms as if this would concern methodological issues only. What the role of the economist is (or should be) in advice-giving and what constraints should be self-imposed in that exercise are on a par with the kinds of professional constraints embodied in the Hippocratic oath. They are essentially claims concerning *professional ethics* -- neither regular ethical nor methodological norms. Part of the confusion in Buchanan’s approach arises, we think, from a failure to distinguish clearly between ethical, methodological and professional norms. Due to this failure, he is inclined to treat professional norms as if they were methodological, simply because they arise professionally, and not from the doctors’ private ethical judgments. Of course, within the domain of professional norms, there remains the distinction between various possible contents of such norms: whether in particular the norms require partiality for the ends of particular advisees, or are of a more universalist character. That latter question may be a professional issue: it may be one aspect of “what economists should do”. But it is essentially ethical, not methodological!

## Particularist vs. universalist individualism

To the extent that they are regarded as ‘*sovereign*’, citizens are under no obligation to respect the ends and choices of *other* citizens in a value-neutral way. On the contrary, “the normative premise that individuals are the ultimate sovereigns in matters of social organization, that individuals are the beings who are entitled to choose the organizational-institutional structures under which they will live” (Buchanan 1991/1999, vol.1, 288) entitles citizens to be partisan for their own *particular* aims, ends or values; and the choices they make in pursuit of their personal ends. Treated as *sovereign* individuals, citizens cannot in this role be taken as obliged to adopt universalist contractarian ends (such a restriction would be a flagrant violation of the ordinary concept of sovereignty). If the economist is living up to Buchanan’s maxim not “to play at being God” in her advisory role, she has to respect what sovereign citizens as a *matter of fact* want and choose.

If the term ‘sovereign’ is used in its conventional sense, then, if citizens themselves choose “to play at being God” (in the sense of imposing their aims and values on non-consenting others), is this not what the economic advisor has to respect according to the norms of her advisory role? If the citizen is willing to make choices that disrespect ends of *other* citizens, ends that conflict with that citizen’s own, then *this* is the end ‘given’ by him for his economist-advisor to pursue.^13^ If as a matter of contingent fact, an action serves their particular ends and if particular citizens can get their way (or ‘get away with it’) the economic advisor seems – as scientific advisor – under a role obligation to provide advice concerning how individuals can within empirical feasibility constraints *impose their particular ends on others* if they want to.^14^ According to the role-obligations of an economic advisor, as Buchanan specifies them, the ‘economic philosopher’ is not entitled to restrict advice to means that serve the ends of citizens other than those he is advising at the time and certainly not of all citizens.^15^

Buchanan does not clearly state whether he thinks of an economic advisor as addressing particular citizens separately with a tailor-made ‘ad personam’ proposal on the one hand, or, on the other, thinks of a representative individual or the collectivity of all individual citizens as addressees. A rather typically ambiguous statement expressive of Buchanan’s “normative individualism” is the following:

“If individuals are considered the ultimate sovereigns, it follows directly that they are the addressees of all proposals and arguments concerning constitutional-institutional issues. Arguments that involve reliance on experts in certain areas of choice must be addressed to individuals, as sovereigns, and it is individuals’ choice in deferring to experts-agents that legitimizes the potential role of the latter…” (Buchanan 1991/1999, vol.1, 288–289).

In his endorsement of what he chooses to call “normative individualism”, Buchanan does not distinguish between *particularist normative individualism*, which demands respect for each citizen separately as a sovereign *whatever* the ends of the *particular citizen* may be and *universalist normative individualism*, which (in its allegedly ‘methodological’ incarnation) demands to give advice that is *respectful of the ends of all citizens*.

The failure to make the preceding elementary distinction does not amount merely to a harmless conceptual ambiguity. On the contrary, it directly induces a failure to distinguish between a particularistic perspective which focuses on the aims ends or values the addressee *as a matter of fact* pursues and a universalistic means-to-given-ends perspective which focuses on ‘universalizable ends’ (and even *contrary to factually* pursued ends). The crucial dividing line between putatively value-neutral advice and ethics-based universalist moral advice would then run *through* economic philosophy rather than separating economic philosophy from moral philosophy.^16^ This would be conceptually possible but contradict two of the few established principles of *philosophical* methodology: the precept to eliminate conceptual distinctions which merely appear to be categorical but are not, on the one hand; and, on the other, to insist on using separate concepts if categorically important distinctions are involved.

To apply this to the preceding distinction between particularistic and universalistic arguments consider an analogous example of triumphant simplicity: somebody may fully agree that each person has an interest in securing her own survival but at the same time can perfectly consistently insist that she happens not to share the end that the survival interests of each and everybody be (universally) protected. As a matter of empirical fact, the protection of her own survival interest may not depend on protecting the survival interests of other or all individuals. It is in no way guaranteed that a universal interest of each on the object level gives rise to a particular interest *of* each that the interest be universally protected *for* each (on the level of protecting object-level interests). Obviously, there may as a matter of contingent empirical fact be a technology by which the ends of some can be better furthered at the expense of others.^17^

The preceding seems so obvious that it must appear implausible that Buchanan and his contractarian followers do not make the distinction between a particularistic and a universalistic conception as relevant sub-categories of what they call “normative individualism”. Yet, it seems that they do indeed inadvertently mix up the case where all individuals separately demand something for themselves, with the case where all demand that that something be universally provided such that every individual receive that something.^18^

Not to make the conceptual distinction between particularism and universalism sufficiently clearly is, we think, a methodological sin of Buchanan. It seems that the substantive ethical ideals of interpersonal respect seduce him and his followers to commit the intellectual sin of confounding norms of a categorically different kind and status with each other. Yet, good intentions excuse but do not justify spreading confusion.

In any event, it is notable that Buchanan focuses his remarks about advice-giving on broad constitutional/institutional matters — matters which are themselves typically decided collectively, and within the Buchanan scheme, under presumed conditions of substantial uncertainty about the particular circumstances the individual will be in when it comes to ‘in-period’ choices. This focus on constitutional advice-giving backgrounds the possibility that individuals might seek advice from economists on particular policies — something that economists have rarely shown any reluctance to provide, but where the different interests of particular individuals are more salient. Buchanan’s focus here seems deliberate. It is chosen precisely to suppress the differences between individual and generalised interests. However, to the extent that the ‘constitutional focus’ is chosen for essentially ethical reasons (precisely because that focus is congenial to Buchanan’s prior contractarian commitments), it seems to us that it simply involves playing God at a more subtle (and arguably misleading) level. There is on the face of things nothing in the methodology of economics that requires the economist, if asked, to refrain from giving advice on particular policy issues; and we cannot rule out the possibility that at least some advisees will be mainly interested in how those policies are likely to affect their individual interests.^19^

One might also distinguish between the general advice that economists may give to individuals — either in opinion pieces in the local press or over the dinner party table — as a matter of essentially gratuitous ‘expert opinion’ on the one hand; and the professional advice that is entailed in the employment contract that an economist might have with her employer. Many economists, after all, work for private companies — and it might be presumed that their contractual obligations involve providing information on actions (including political actions) that are in the best interests of the company that employs them (but not necessarily in the interest of a broader public). In short, if there were a serious interest in “what economists should do”, one might think that in their advice-giving roles (of which there are many, varied, possibilities) some attention might be given to the economist-advisor’s particular location in the professional landscape. Buchanan’s treatment does not seem to allow for such niceties.

## Conclusion

There would not be much of a problem if a subgroup of economists would openly declare: that they are Buchantian contractarians, that this is an explicitly ethical position and that in that sense they are operating outside ‘predictive’ economic science. They would then without (self-)deception compete with other disciplinary groups for attention, funding etc. Their research would belong to a special branch of moral philosophy or what traditionally has been called applied normative ethics. This is altogether legitimate. What would be illegitimate for anybody who values transparency is the effort to camouflage the nature of that enterprise by dressing it up as if it were an exercise in evidence-based value-neutral science and in line with ordinary scientific methodology.

Economists should not behave like groups of homoeopathic medical doctors who want to have it both ways: sail under the flag of regular evidence-based medicine, sharing its reputation as (modestly) reliable ‘school medicine’ but at the same time not comply with the standards of clinical and evidence-based school medicine. When adherents of ‘alternative medicine’ call what is decidedly meant as opposed to school medicine by the same name, this amounts in practical terms to a deception of potential clients. Though this deception may be based on a self-deception and in this sense be less egregious than sheer manipulation, it seems illegitimate according to common sense standards of everyday honest practice to stick to erroneous self-classification if that can be avoided by consistent application of a transparent terminology.

In case of alternative medicine, it remains fully legitimate to declare openly what the alternative discipline is all about and then compete with school medicine. As Wilhelm von Humboldt (1851/1993) argued, practitioners of alternative medicine could even be treated as entitled to the title of a ‘medical doctor’ if this is the term used for the healing profession generally. But they are not entitled to masquerade as doctors *certified* according to the standards of school medicine (and the less so of modern evidence-based medicine).^20^

Likewise, one can legitimately declare oneself to be a contractarian economist or a classical liberal economist or what have you, as long as this declaration is not made in ways that blur the relevant distinctions between economics as a predictive science and economics as a branch of moral philosophy.

It is at the very least unhelpful to confuse methodological norms with ethical ones; and by this latter category we mean to include professional as well as personal ethical norms. This is not to claim that value-free means/ends claims are the only game in the town of applied economics. It is simply to insist that Buchantian contractarians cannot hide behind any suggestion that their enterprise is value-neutral economic philosophy rather than a normative/ethical enterprise.

Unlike Buchantianism, Hans Albert’s critical rationalist economic philosophy is value-neutral. It does not claim to provide a justification for incorporating substantive moral/political values into economics as a discipline. This does not rule out that the present authors, Buchanan and his followers might all be adherents of ideals of classical liberalism and the institutions of WEIRD societies (Western, Educated, Industrialized, Rich, Democratic ones).^21^ Whatever the internal quibbles amongst such adherents of open societies may be, they definitely concur in the objective of sustaining such societies. But this is a commitment that cannot reasonably claim to float free of substantive ethical values. As far as methodological values are concerned, we ‘predict’ that the ‘Albertian’ focus on value-neutral technological arguments, on instrumentally rational thinking in terms of exploring means to potential ends that need to be exogenously given, is itself a technology that as a matter of empirical fact contributes to the desired sustainability of WEIRD societies. As a prediction of constitutional economics, it is based on empirical hypotheses on how inclusive political institutions work and stabilize themselves. On the moral philosophical issue of the ultimate ethical legitimacy of Buchantian contractarianism such an Albertian technological constitutional economics remains silent and as value-neutral science necessarily so.

* Max Albert’s helpful critical comments are gratefully acknowledged and the follow up contribution to Homo oeconomicus in which we have much more to say on the basic contractarian ideal of politics as exchange and its role in the approaches of Buchanan, Sugden and Vanberg is announced.

## Footnotes

1 A social technology is a kind of blueprint of possible institutional solutions. It is based on tested scientific knowledge of ‘cause and effect’ derived from economics as a predictive science. As conceptualized by Hans Albert technologies describe mechanisms that have predictable properties when implemented. In this sense technologies make falsifiable claims about practical cause and effect relations that can be tested as (other) scientific propositions; see (Albert, 1985).

2 We are alluding here to the subtitle of Hume (1739/1978).

3 More traditionally put, technological thinking is what Hume and Smith understood by philosophy — which in their account was mostly coincident with what we nowadays understand by science. In this tradition, economic philosophy and economic science have perhaps more in common than is presumed in many recent philosophical accounts that sought to distinguish the two.

4 “Embroiled in Controversies” (“*In Kontorversen verstrickt*”) is the descriptively accurate title of Hans Albert’s (2007) autobiography and we seek to follow his example here.

5 Buchanan and his followers -- we are thinking here in particular of Sugden (2018a, 2018b) and Vanberg (2021) -- seem sometimes to assume that imposing normative claims on citizens is avoided if advice is tendered to ordinary *citizens* rather than to ‘benevolent despots’ as sovereigns but obviously there may be inter-individual conflict between sovereigns.

6 McIntyre is an adversary of the “enlightenment paradigm” (Wootton, 2018, Chap. 1) who understood well what he was attacking. In launching his extended attack on the paradigm McIntyre aptly characterizes it as follows: “Reason is calculative, it can assess truths of fact and mathematical relations but nothing more. In the realm of practice therefore it can speak only of means. About ends it must be silent.” (McIntyre, 1984, 54).

7 See for a succinct and balanced survey of Buchanan’s major criticisms Sugden (2018a).

8 Wootton (2016) is a brilliant critical rationalist account of “the invention of science” and the value-neutral respect for ‘facts’ as an expression of what Hans Albert endorses as ‘critical realism’.

9 Hans Albert and his son Max have sought to extend institutional economics in particular to the institutions and practices of economic science itself (Albert, 2006, 2010) but think of economics as a broad church covering institutional and behavioural analyses.

10 The aim is to confer powers to make their own choices on individuals equally. The implementation of contractual exchange-enabling institutions being the paradigm example; see Buchanan (1964/1999, vol. 1), Sugden (2018a, 2018b), Vanberg (2021 in this volume Albert@100).

11 In a coherent value-neutral interpretation of the advisor role individuals with conflicting ends can receive purely technological advice on how to get their way against other individuals who get advice on how they might get their possibly conflicting way. How to render such theory-based advice ‘absorbable’ – basically be known publicly without undermining its technological suitability – is one of the deeper problems at root of the development of game theory and the reasoning about knowledge in games; see instructively on this Leonard (2010).

12 On the concept of a case as a *type* of situation rather than a ‘token’, see Alchurron and Bulygin (1971) whose approach naturally extends from law to other practical normative systems.

13 It is meant to be guiding the citizen’s agent in the search for adequate means to reach the citizen-principal’s ends.

14 To be entirely clear on this, citizens who have the power to or can impose their particular will on others are entitled to do so according to Buchanan’s own requirement that the individual citizen – rather than some community of citizens – is sovereign (i.e. ‘legibus absolutus’ and not subject to some ‘obligatio naturalis’). It is Buchanan himself who follows Hobbes in projecting the Bodinian (1576/1992) notion of sovereignty on the individual actor as originally endowed with a “right to everything” – a concept of a ‘right’ which boils down to the absence of *any* normative obligation to refrain from seizing any opportunity or using any “power to achieve any apparent good” (Hobbes 1651/1968, initial sentence of § 10).

15 Unless, of course, as a matter-of-fact citizens themselves ask for universalist advice; e.g. A asks for what is furthering the ends of A, B, C and so do B and then C. Of course, one could wheel in an auxiliary argument that economists like other citizens are entitled to refuse engaging in a broadly contractual relationship which would require them to provide non-universalist advice. But then general norms of honesty would require that economists would make this restriction transparent to their potential advisees. This is not what Buchanan and his followers do!

16 At least unless *two* sub-concepts of ‘normative individualism’ are categorically distinguished and discussions realigned accordingly.

17 That certain measures make some relatively better off than alternative measures, may also apply to alternative measures that relative to some status quo make all better off since there may be conflicts concerning which of several Pareto-superior moves in a state is chosen.

18 This is not to say, that it may not be desirable and prudent to demand universal provision. Quite to the contrary, those who do want to live under the umbrella of a Western type of liberal democracy in fact must for conceptual reasons desire that the legal order and the sense of justice supporting it (and supported by it and so on…) is universalist with respect to the members of the relevant community. We are saying only that this is a distinct substantive judgment, distinct that is, of the wish of individuals to get their own way even if detrimental to others.

19 The fact that individuals exercise very little influence on policy determination seems likely to induce some changes in their motivations for participating in collective decisions on policy matters — an issue that plays a major role in the ‘expressive’ account of voting (see for example Brennan & Lomasky 1993).

20 Again it deserves to be acknowledged that even today, most established clinical practices are not evidence-based in any standardized sense.

21 Of course, we are alluding to Henrich (2020).
